# Neuroprotective Herbs and Foods from Different Traditional Medicines and Diets 

**DOI:** 10.3390/molecules15053517

**Published:** 2010-05-14

**Authors:** Marcello Iriti, Sara Vitalini, Gelsomina Fico, Franco Faoro

**Affiliations:** 1 Dipartimento di Produzione Vegetale, Università degli Studi di Milano, Milano, Italy; 2 Dipartimento Agroalimentare, CNR-IVV, Milano, Italy; E-Mail franco.faoro@unimi.it (F.F.); 3 Orto Botanico ‘GE Ghirardi’, Università degli Studi di Milano, Toscolano Maderno, Brescia, Italy; E-Mail: sara.vitalini@unimi.it (S.V.); 4 Dipartimento di Biologia, Università degli Studi di Milano, Milano, Italy; E-Mail: gelsomina.fico@unimi.it (G.F.)

**Keywords:** Ayurvedic medicine, Mediterranean diet, neurodegenerative diseases, nutritional therapy, phytotherapy, Traditional Chinese Medicine

## Abstract

Plant secondary metabolites include an array of bioactive constituents form both medicinal and food plants able to improve human health. The exposure to these phytochemicals, including phenylpropanoids, isoprenoids and alkaloids, through correct dietary habits, may promote health benefits, protecting against the chronic degenerative disorders mainly seen in Western industrialized countries, such as cancer, cardiovascular and neurodegenerative diseases. In this review, we briefly deal with some plant foods and herbs of traditional medicines and diets, focusing on their neuroprotective active components. Because oxidative stress and neuroinflammation resulting from neuroglial activation, at the level of neurons, microglial cells and astrocytes, are key factors in the etiopathogenesis of both neurodegenerative and neurological diseases, emphasis will be placed on the antioxidant and anti-inflammatory activity exerted by specific molecules present in food plants or in remedies prescribed by herbal medicines.

## 1. Introduction

Neuroprotection refers to the strategies and relative mechanisms able to defend the central nervous system (CNS) against neuronal injury due to both acute (e.g. stroke or trauma) and chronic neurodegenerative disorders (e.g. Alzheimer's disease, AD, and Parkinson's disease, PD). Among these strategies, herbal medicine may represent a valuable resource in prevention rather than in therapy of some CNS diseases, in association with a healthy lifestyle including correct dietary habits and moderate physical activity. As complementary and alternative therapy, herbal medicine, or simply phytotherapy, refers to the medical use of plant organs (leaves, stems, roots, flowers, fruits and seeds) for their curative properties. Generally, herbal products contain complex mixtures of active components (phytochemicals), including phenylpropanoids, isoprenoids and alkaloids, and it is often difficult to determine which component(s) of the herb(s) has biological activity [1,2,3,4].

Nutritional therapy is a healing system using functional foods and nutraceuticals as therapeutics. This complementary therapy is based on the assumption that food is not only a source of nutrients and energy, but can also provide health benefits. In particular, the reported health-promoting effects of plant foods and beverages can be ascribed to the numerous bioactive chemicals present in plant tissues and, consequently, occurring in foods. Consumed as part of a normal diet, plant foods are thus not only a source of nutrients and energy, but may additionally provide health benefits beyond basic nutritional functions, by virtue of their dietary therapeutics (phytochemicals) [5,6,7,8].

In this survey, we briefly introduce neurodegenerative diseases, AD and PD in particular, with emphasis on the preventive strategies represented by herbal medicine and nutritional therapy. We provide an ethnobiological approach, focusing on plant foods and medicinal herbs used by different traditional medicines and diets and relevant for some of their neuroprotective components.

## 2. Neurodegenerative diseases

A neurodegenerative disease is defined as a deterioration, often irreversible, of the intellectual and cognitive faculties and it is generally associated with ageing and/or AD, PD, stroke. 

### 2.1. Ageing and oxidative stress

With the increase of ageing population, neurological diseases represent a relevant health problem. *Sensu lato*, an age-dependent degenerative disorder represents a condition in which the function and/or structure of affected tissues or organs experience progressively deterioration over time, such as cardiovascular and neurodegenerative diseases, immune system (immunosenescence) and skeleton muscle decline (sarcopenia). Ageing is a complex physiological process that involves both morphological and biochemical changes occurring, with the passage of time, in single cells and the whole organism. Among the many theories proposed to explain the mechanisms of ageing at the molecular level, the oxidative stress or free radical hypothesis has received wide support. At biochemical level, oxidative stress can be defined as a disturbance in the cell oxidation/reduction (redox) status, leading to the production of partially reduced oxygen intermediates, more reactive than molecular oxygen in its ground state, including both radical and nonradical forms collectively termed as reactive oxygen species (ROS). Therefore, ROS production and oxidative damage to biomacromolecules (nucleic acids, lipids and proteins) can represent a suitable environment for the development of age-related diseases [9].

Neurodegeneration is a process involved in both neuropathological conditions and brain ageing. Although the brain accounts for less than 2% of the body weight, it consumes about 20% of the oxygen available through respiration. Therefore, because of its high oxygen demand, the brain is the most susceptible organ to oxidative damage [10,11,12]. Additionally, the high amount of polyunsaturated fatty acids (PUFAs) present in neuronal membranes makes the brain tissues particularly susceptible to lipid peroxidation reactions, resulting in the formation of cytotoxic aldehydes, such as malondialdhyde (MDA) and 4-hydroxynonenal (HNE) [13].

To protect vulnerable targets, organisms have evolved sophisticated strategies, collectively termed antioxidant defences, that counteract the imbalance of the cell redox homeostasis and keep the ROS levels under the cytotoxic threshold [14]. Antioxidant defences also comprise (plant) vitamins and phytochemicals, *i.e.* nonenzymatic scavengers abundant in food and medicinal plants and introduced by diets [15,16,17,18]. Any compound capable of quenching ROS, without itself undergoing conversion to a destructive radical species, can be considered as an antioxidant, as in the case of dietary phytochemicals [19,20].

### 2.2. Alzheimer’s (AD) and Parkinson’s (PD) diseases

Amongst a variety of neurodegenerative diseases, Alzheimer's disease is the most prevalent and devastating disorder and the first cause of institutionalisation in the elderly population. Clinical signs of AD are characterized by progressive and irreversible memory deficits, cognitive deterioration and personality changes, usually with an onset after 65 years of age. Memory impairment appears in the early stage of the disease, and motor and sensory functions are not affected until later stages. Parkinson's disease is the second most common ageing-related neurodegenerative diseases that can greatly impair quality of life. As opposed to cognitive deficits of AD, PD is a movement disorder, whose classical signs include resting tremors, bradykinesia, extrapyramidal rigidity and loss of postural reflexes such as disturbance in walking or equilibrium. The consequence of these diseases are also very significant in terms of the cost of caring for patients [21,22].

#### 2.2.1. Epidemiology of AD and PD

Incidence rates of neurodegenerative diseases such as AD and PD increase exponentially with age. According to the World Health Organization (WHO), neurodegenerative diseases will become the world’s second leading cause of death by the middle of the century, overtaking cancer [23]. In 2000, there were 4.5 million Americans diagnosed with AD, with an annual estimated cost of $100 billion [24]. Presently, it is predicted that by 2050 the number of AD patients in the US population could range from 11 to 16 million if no cure or preventive measure is found [25]. AD is the most common cause of dementia in the elderly and accounts for 60–80% of cases. According to the Global Burden of Disease Study, dementia and other neurodegenerative disorders will be, by 2020, the eighth cause of disease burden for developed regions [26]. In Europe, AD shows a prevalence of 0.4% in women and 0.3% in men aged 60–69 years [27].

PD affects approximately 1% of population aged 65–69 years and the prevalence increases to 3% in the 80 year old or above group [28]. Its incidence is approximately 1.5 times higher in men than woman at all ages [29]. It is estimated that in Western Europe, by 2030, the number of PD cases will double from 4.5 to 9 million [30]. The prevalence of PD varies in different ethnic and geographic groups [31]. For instance, a north-south geographic gradient has been observed for PD, with prevalence increasing with latitude in both hemispheres, as compared with the equatorial zones. Furthermore, white populations appear more at risk than black subjects [32]. 

#### 2.2.2. Neuropathology of AD and PD

Although AD and PD largely differ in their clinical symptoms and disease course, both disorders are basically provoked by a progressive loss of neurons in different neuronal systems. Furthermore, as for most neurodegenerative diseases, they are characterized by the aggregation of intracellular proteins [33]. The presence of extracellular senile plaques and intracellular accumulation of neurofibrillary tangles (NFTs) in brain tissues of affected patients have been identified as histopathologic alterations of AD. Senile plaques are composed of fibrillar amyloid β (Aβ) peptides produced by cleavage of the Aβ precursor protein (APP), whereas NFTs consist of hyperphosphorylated microtubule associated tau protein (Pτ). Selective neuronal loss is particularly severe in specific cerebral areas: the neocortex, hippocampus, limbic system and subcortical areas [34].

PD is characterized by the selective degeneration of dapaminergic neurons located in the pars compacta of the substantia nigra. One of the main neuropathological hallmarks of PD is the aggregation of the intracellular protein α-synuclein, to form intracytoplasmic inclusions (Lewy bodies) in these neurons [35].

#### 2.2.3. Etiopathogenesis of AD and PD

The etiology of neurodegenerative diseases is multifactorial, with a complex combination of genetic and non-genetic components. Most neurodegenerative diseases occur sporadically, arising from the interaction among environmental factors and genetic susceptibility. Only a small minority of cases are of purely genetic origin, due to a mutation of genes encoding for the abnormally aggregating proteins [36]. Age is the only reliable risk factor for the sporadic forms, differently from familial forms that can exceptionally affect young subjects [37].

Currently, oxidative damage is believed to be one of the leading cause of neuronal degeneration in both AD and PD [38,39,40,41,42]. Though the exact mechanisms of AD are still unknown, several lines of evidence suggest that oxidative stress is implicated in Aβ-induced neurotoxicity, besides apoptosis and inflammation, two processes strictly related to ROS overproduction [43,44,45]. Moreover, the aggregation and toxicity of the Aβ peptides involve transition metals. Data suggest a close relationship between cerebral biometal (Fe, Cu, Zn) deregulation and AD pathology, with redox-active metals and Aβ peptides that interact to elevate oxidative stress in brain tissues [46,47]. Mitochondria, a main source of ROS since their aerobic metabolism, represent a main biochemical target of AD. Damaged mitochondria are less efficient producers of energy in form of ATP and more efficient producers of ROS, thus exacerbating oxidative stress [48].

In PD, all the mechanisms involved in selective degeneration of dopaminergic neurons in the nigrostriatal system are still unknown, though evidence suggests the involvement of ROS. Oxidative stress may arise from the metabolism of dopamine with the production of potentially harmful free radical species [49]. Compared to the rest of brain, the subtantia nigra pars compacta is exposed to a higher rate of ROS formation and to higher levels of oxidative stress. The reason for this is not clear yet, but may be related to the energy metabolism of these cells or to their high content of dopamine [50].

#### 2.2.4. Current pharmacological intervention for AD and PD

There is no cure for neurological diseases and only symptomatic treatments are available. Current therapies for AD and PD mainly provide symptomatic improvement by replacing the levels or controlling the metabolism of neurotransmitters involved in the diseases, to restore their imbalance. Cholinesterase inhibitors are the earliest developed and still the first line prescript drug available for patients with mild to moderate AD. By inhibiting the hydrolysis of acetylcholine in the synaptic cleft, these drugs restore the levels of the neurotransmitter in the affected neurons of AD patients [51,52]. For moderate to severe cases, memantine has been approved. This drug acts as a specific, non-competitive *N*-methyl-D-aspartate (NMDA) receptor antagonist able to counteract the excitotoxicity of glutamate, the major excitatory neurotransmitter in the brain [53,54]. Anti-inflammatory and antioxidant therapies have been also proposed as possible preventive strategies, though, at present, conclusive data are not available [55,56].

Current therapeutic approach for the symptomatic treatment of PD includes levodopa (L-DOPA) as the most effective drug. After administration, levodopa is converted to dopamine, thus replenishing the diminished dopamine levels in affected tissues [57,58].

### 2.3. Acute cerebrovascular attack (stroke)

This is an ischemia (a reduction or a blockage of oxygen and glucose supply) generally caused by thrombosis or haemorrhage which blocks the blood flow to a specific brain region. In particular, the occlusion of a cerebral vessel due to a blood clot (thrombus) discharged from the surface of an atherosclerotic lesion may cause an ischemic stroke, whereas an intracranial or intracerebral haemorrhage, commonly caused by hypertension, may lead to a haemorrhagic stroke [59,60]. 

Stroke is currently the second leading cause of death in industrialized countries, ranking after heart disease and before cancer, and a major cause of permanent disability [61]. Interestingly, about 25% of patients develop dementia within three months after a stroke [62,63]. In fact, both Aβ and APP are deposited in cortical and subcortical brain areas of non-dementia patients following stroke [64].

## 3. Traditional Chinese Medicine

Traditional Chinese Medicine (TCM) is one of the world’s oldest documented medical systems based on herbal medicines. It has been estimated that about 20% of the plant species listed in Flora Sinica are used as medicaments [65]. Typical TCM concepts are *Yin*, *Yang *and *Qi*, components of ‘vital energy’ difficult to translate into Western medical terms. Unlike the concept adopted in Western medicine, ‘vital energy’ in TCM is a term for collectively describing both the mental and physical energy. It is believed to be essential for growth, daily activities, reproduction, cognitive functions and unbalance of *Yin* and *Yang* breaks body’s harmony predisposing to diseases. To restore this harmony is the principle for treating illnesses. In Chinese tradition, ageing is considered a progressive decline of vital energy in our body, and anti-ageing herbs are able to correct the imbalance of vital energy components [66,67]. In the ancient TCM book ‘Yellow Emperor’s Canon of Internal Medicine’, appropriate lifestyles are described to preserve vital energy in our body, such as optimal diet, moderate exercise and reduction of both mental and physical stress, able to increase lifespan beyond 100 years of age [68]. According to the tradition, the Yellow Emperor established herbal medicine about 5,500 years ago, assaying hundreds of plants to discover medicinal herbs [69]. Despite the completely different basic philosophical principles, the ancient, but still vital, TCM has represented a therapeutic wisdom for Western medicine in recent years, as in case of anti-ageing herbs [70]. In the following section, *Ginkgo biloba* L., *Panax ginseng* C.A. Mayer and *Scutellaria baicalensis* Georgi, herbs mostly involved in neuroprotection, will be briefly described.

### 3.1. *Ginkgo biloba* L. (Yín Xìng or Yín Hsìng)

**Figure 1:**
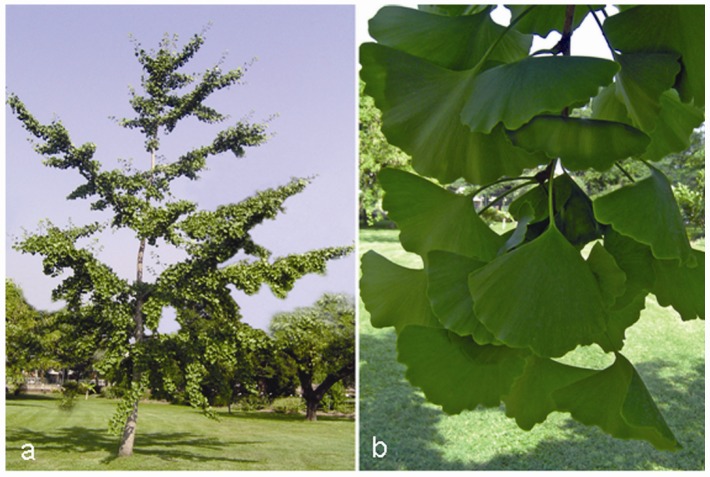
*Ginkgo biloba* L. tree (a) and its typical fan-shaped leaves (b).

From the Chinese term *Ginkyo* meaning silver apricot, due to its yellow seeds, it is a tall and ancient tree (Figure 1a) with a remarkable long life span of more than 4,000 years due, at least in part, to its high tolerance to abiotic/environmental stresses and resistance to pathogen infections. This species, belonging to the Ginkgoaceae family, is considered a ‘living fossil’ because of its primitive characters and absence of close living relatives [71]. The medical use of this plant can be traced back to approximately 5,000 years to the origins of TCM. Modern Chinese pharmacopoeia introduced *Ginkgo* leaves, typically fan-shaped (Figure 1b), as treatment for vascular insufficiency and to improve longevity, whereas seeds are used as astringent for the lung to treat illnesses like asthma and chronic bronchitis [72].

**Figure 2:**
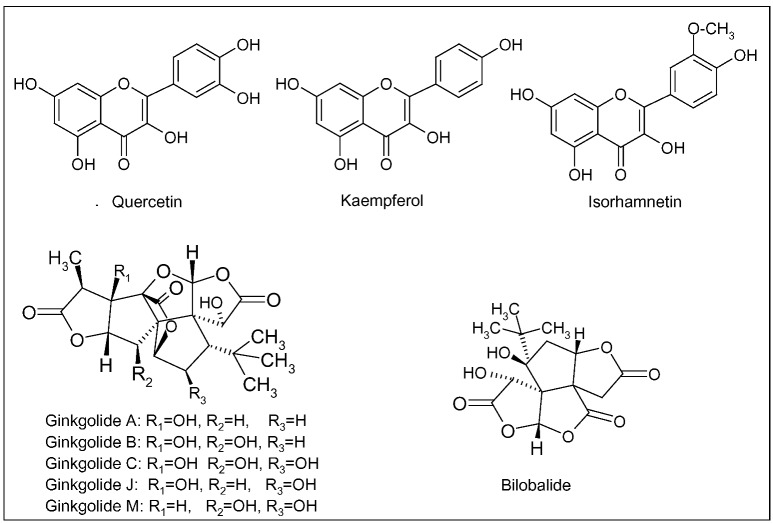
Main flavonoids (quercetin, kaempferol and isorhamnetin), ginkgolides (A, B, C, J, M) and bilobalide of *Ginkgo biloba* L.

Since 1965, German physicians have prescribed *G. biloba* for the treatment of cognitive dysfunction, dementia and AD. In the early 1970s, the standardized extract of *G. biloba* leaves (EGb 761) was registered and, since then, patented products were developed and commercialized [73]. Presently, standardized extracts are widely prescribed in Europe and US for the symptomatic treatment of AD and cerebral insufficiency (a nonspecific age-related deterioration of mental functions), and for the improvement of cerebral blood flow and memory [74]. EGb 761 contains 24% of flavonoids and 6% of terpenic lactones, giving this extract its unique polyvalent pharmacological action. The flavonoid fraction is mainly composed of three flavonols, quercetin, keampferol and isorhamnetin, whereas terpenic derivatives are represented by diterpenic lactones, the ginkgolides A, B, C, J and M, and a sesquiterpenic trilactone, the bilobalide (Figure 2) [74]. Ginkgolides are antagonist of platelet-activating factor (PAF), able to reduce platelet activation and aggregation, thus improving blood circulation [75]. Bilobalide can reduce damage caused by global brain ischemia and excitotoxicity-induced neuronal death [76].

In general, the neuroprotection exerted by EGb 761 is due to the combination of antioxidative, anti-amyloidogenic and antiapoptotic activities, by virtue of the blend of its bioactive phytochemicals [77,78,79,80,81]. The efficacy of EGb 761 has been assessed by different clinical studies. In a randomized, double blind, placebo-controlled trial, patients of the treatment groups received over a 24-week period an oral daily dose of 160 mg EGb 761 or 5 mg donepezil (a cholinesterase inhibitor), whereas the control group was treated with a placebo. According to the primary outcome measures (Syndrome Kurz Test, SKT, Mini-Mental State Examination, MMSE, and Clinical Global Impression, CGI), the study showed that both EGb 761 and donepezil were more effective than the placebo in improving the cognitive function of patients with mild to moderate AD, whereas no statistical difference was reported between treatments [82]. In a meta-analysis reviewing many randomized, double blind, placebo-controlled clinical studies, patients diagnosed with AD received 120 to 240 mg/day of EGb 761 for 3 to 6 months. A standardized neuropsychological scale, the Alzheimer’s Disease Assessment Scale Cognitive subscale (ADAS-Cog), was used to evaluate the efficacy of treatment on cognitive function. It was observed a modest, but significant, effect translated into 3% improvement on the primary outcome (ADAS-Cog) in treatment group [83]. Similar results were reported later in a cohort of woman aged 75 years and older [84]. Currently, two phase III clinical trials, the GEM (Ginkgo Evaluation of Memory) study in US and the GuidAge study in France, focus on the evaluation of EGb 761 efficacy in the prevention of AD in more than 3,000 patients/study older than 70 years. Both studies were randomized, double blind, placebo-controlled trials [85,86]. In GEM study, EGb 761 was administered in a dose of 120 mg twice daily and the incidence of all-cause dementia was used as primary outcome. Secondary outcome included the rate of cognitive decline, the incidence of cardio- and cerebrovascular events and mortality [85,87]. In GuidAge study, the efficacy of 240 mg daily of EGb 761 was evaluated, with the incidence of AD during a 5-year follow up period as primary outcome [87]. The latter study is the largest clinical trial carried out in Europe on the prevention of AD and its final results will be available in 2010 [87].

### 3.2. *Panax ginseng* C.A. Mayer

**Figure 3:**
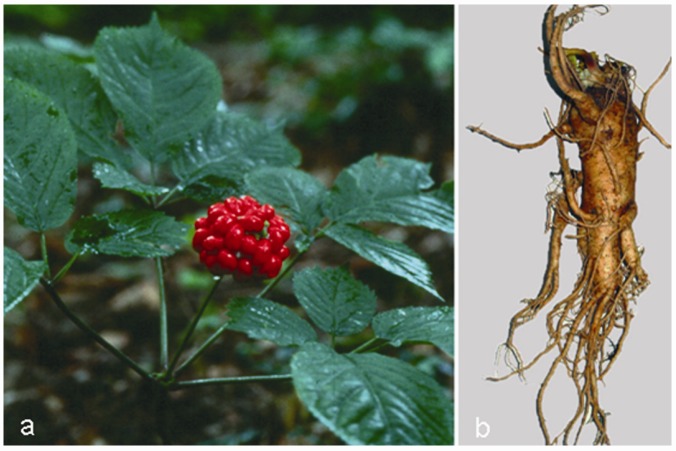
*Panax ginseng* C.A. Meyer: aerial parts (a) and root (b).

Ginseng (from the Chinese *rènshēn* = man root, which refers to the root shape resembling the leg of a man), one of the most widely used herbs in TCM for boosting Qi (energy), is another anti-ageing herbs, employed for thousands of years as a tonic and revitalizing agent [88]. Ginseng refers to a group of several species within the *Panax* (from the Greek *pan* = all, and *akèia* = cure) genus and the family of Araliaceae growing in north-eastern Asia, with *P. ginseng *(Figure 3a), or Asian ginseng, among the most commonly used species also in Korean traditional medicine and Kampo (Japanese traditional medicine). Other important species are Vietnamese ginseng (*P. vietnamensis* Ha et Grushv.), Siberian ginseng (*Eleutherococcus senticosus* Maxim., which is not a true ginseng) and American ginseng (*P. quinquefolius* L.) [89].

**Figure 4:**
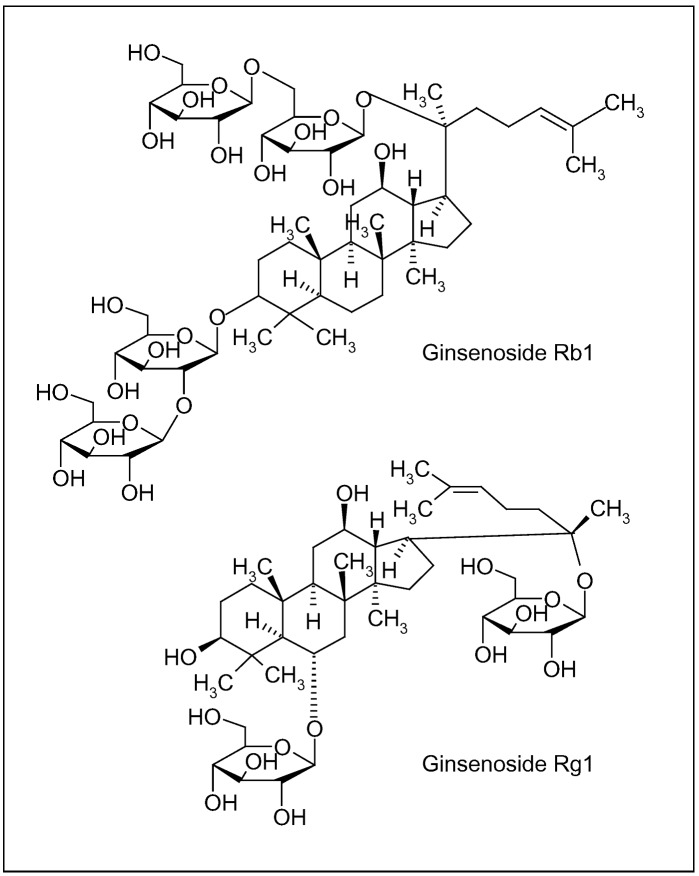
Ginsenosides of *Panax ginseng* C.A. Meyer root; Rb1 and Rg1 are characterized by different aglycones, protopanaxadiol and propanaxatriol, respectively.

Ginseng root (Figure 3b), characterized by the presence of ginsenosides (triterpenic saponin complexes) (Figure 4), is considered an adaptogenic herb, able to increase the body’s resistance to stress, trauma, anxiety and fatigue by modulating the immune function. Furthermore, it improves memory, learning performance, motor activity and, as aphrodisiac, ginseng can be applied to patients with sexual dysfunction. Depending on their aglycone, ginsenosides are often divided into two groups: Rb1 group (Rb1, Rb2, Rc and Rd) and Rg1 group (Re, Rf, Rg1 and Rg2), characterized by the presence of protopanaxadiol and propanaxatriol, respectively (Figure 4) [90,91,92].

Ginseng may provide protection against neurodegeneration by multiple mechanisms. In different experimental models of AD, it attenuates Aβ- and glutamate-induced toxicity, enhances clearance of Aβ by stimulating the phagocytic activity of microglia and promotes neuron survival, increasing the levels of neurotrophic factors [93,94,95,96,97]. These data show that ginseng components act on different stages of the neurodegenerative disease, thus suggesting that multi-target properties are optimal for a neuroprotective herb.

Human studies have investigated the efficacy of ginseng too. In a clinical trial, patients aged 50 years or older with mild to moderate AD dementia were randomized into three groups. Two treatment groups received an oral daily dose of 9 or 4.5 g of Korean red ginseng (similar to Chinese ginseng) for 12 weeks. The primary outcome measures were MMSE, ADAS and Clinical Dementia Rating (CDR). The high-dose ginseng group showed scores of the outcome measures significantly higher than the control group ones. Differences between low-dose ginseng and placebo were not significant [98].

Apart from AD, ginseng has shown protective effects against PD in several cell culture and animal models. Both ginsenosides and root extracts are able to promote neuronal cell survival by reducing the neurotoxicity induced by toxins or parkinsonism mimetics, such as 1-methyl-4-phenyl-1,2,3,6-tetra-hydropyridine (MPTP) and its active metabolite 1-methyl-4-phenylpyridinium (MPP+) in rodents. These neurotoxins induce oxidative stress and lead to cell death of dopaminergic neurons, as in PD [99,100,101]. In other studies, ginsenosides, besides protecting neuronal cells, have shown neurotrophic effects promoting neurite overgrowth [102,103].

### 3.3. *Scutellaria baicalensis* Georgi (Huáng Qín)

**Figure 5:**
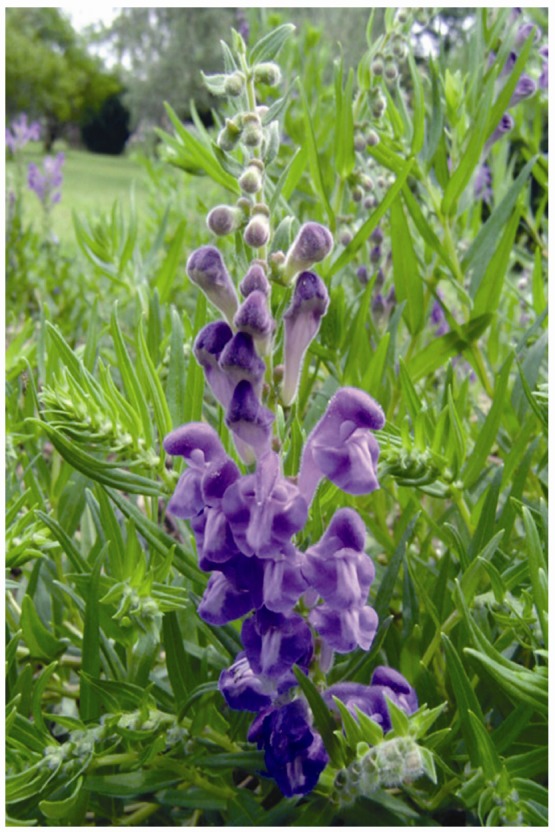
*Scutellaria baicalensis* Georgi aerial parts.

This is a medicinal plant (Figure 5) belonging to the Lamiaceae family and widely used in the oriental (Korean and Chinese) traditional medicine because of its powerful anti-inflammatory and antioxidant activity. 

In Korea, a root extract rich in bioactive components is used to treat diverse (neuro)inflammatory diseases [104,105,106,107]. In CNS, neuroglia plays a critical role in inflammation. Upon inflammatory stimulus (infection, trauma or other), microglial cells, the resident immune effectors (the counterpart of monocytes/macrophages in the periphery), migrate to the site of damaged tissue to defend the organism. In some cases, if their defence responses are not tightly regulated, they may further injury the host tissue by (over)producing ROS and releasing inflammatory mediators, such as cytokines and eicosanoids. Similarly, proliferation and activation astrocytes, a population of glial cells which provide mechanic and metabolic support to neurons, may exacerbate these neurotoxic effects in the inflammation environment [108,109,110].

**Figure 6:**
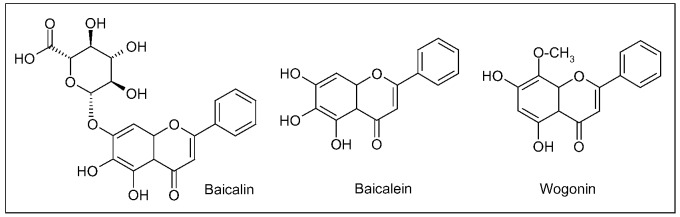
Main flavonoids isolated from *Scutellaria baicalensis* Georgi root.

Three main flavonoids, baicalein (5,6,7-trihydroxyflavone), baicalin (baicalein 7-*O*-glucuronide) and wogonin (5,7-dihydroxy-8-methoxyflavone) (Figure 6), isolated from the root have been assayed for numerous potential benefits [111]. As antioxidant agents, in different cell culture and animal models, these compounds showed to quench ROS, to protect neurons from oxidative damage in cerebral ischemia/reperfusion (I/R) injury, to inhibit lipid peroxidation of neuronal membranes and to prevent excitotoxicity induced by glutamate [112,113,114,115,116,117,118,119,120,121]. 

As an inhibitor of inflammation in the CNS, wogonin suppressed both NO (nitric oxide, a reactive nitrogen species able to increase the cell oxidative burden) production and inducible nitric oxide synthase (iNOS) activation in cultured rat astrocytes [122]. The same *S. baicalensis* metabolite inhibited the inflammatory cascade, in LPS-stimulated, cultured microglia cells, by the suppression of cytokines, such as TNF (tumor necrosis factor)-α and IL (interleukin)-1β, and of the pro-inflammatory transcription factor NF(nuclear factor)-κB [119,123]. NF-κB is widely expressed in tissues of nervous system. It resides in the cytoplasm as inactive form which translocates to the nucleus upon activation. In neurons, NF-κB is activated by various stimuli, such as cytokines, neurotrophic factors, neurotransmitters, and, once in the nucleus, it binds to specific DNA consensus sequences, regulating the transcription of genes involved in immune and inflammatory responses [124]. Inhibition of inflammatory activation of microglia by wogonin reduced cytotoxicity towards co-cultured PC12 neurons, supporting an *in vitro* neuroprotective role of this flavonoid. The efficacy of wogonin was further demonstrated in two experimental brain injury models. In the 4-vessel occlusion model of transient global ischemia, wogonin decreased the death rate of hippocampal neurons, the induction of iNOS and TNF-α in hippocampus, whereas, in the kainate injection model, this flavonoid markedly protected from excitotoxic brain injury [119]. Similarly, baicalein attenuated the NO production by suppressing iNOS induction, in LPS-activated BV-2 mouse microglial cells, besides reducing apoptotic cell death and NF-κB activation [107]. Finally, baicalein and baicalin protected neurons from Aβ-induced toxicity, inhibited fibrillation of α-synuclein and disaggregates existing fibrils [125,126,127].

The amount of baicalin in *Scutellaria* is approximately 10-fold that of baicalein. After oral administration of the aqueous extract of *S. baicalensis*, baicalein, baicalin and wogonin are rapidly absorbed. Baicalin is metabolized into baicalein by bacteria prior to intestinal absorption, the latter metabolite being detected in plasma up to 24 h. In rat, baicalein enters brain crossing BBB and distributes in cortex, hippocampus, striatum, thalamus and brain stem in 20 minutes [128,129,130,131].

## 4. Ayurvedic Medicine

Ayurveda is a very ancient and complex knowledge system including philosophy, religion and science (medicine) applied to daily life. It is based on three fundamental principles or *doshas*, *vata*, *pitta* and *kapha*, that regulate all cellular processes responsible for healthy life. *Vata* governs movements and activities, *pitta* the heath and energy levels, whereas *kapha* regulates growth and structural modification. When these principles get disturbed, because of unfavourable environment or poor diet, the individual starts suffering from some diseases [132]. In India, the history of medicine can be traced to the remote past, with one of the earliest mention of medicinal use of plants reported in Rig Veda, a sacred collection of Sanskrit hymns written between 4,500 and 1,600 BC [133]. Since antiquity, Ayurveda is practiced through its eight specialized branches: *Kayachikitsa* (internal medicine), *Salya* (surgery), *Salakya* (ophtalmology and ENT), *Kaumarbhritya* (pediatrics), *Aagada* (toxicology), *Bhuta Vidya* (psychology), *Rasayana* (rejuvenation) and *Bajikarana* (or *Vajikarana*, sexology) [134]. Among these disciplines, *Rasayana tantra* regards the treatments to improve longevity, memory, to attain youthful appearance, to maintain cognitive performance and physique strength. As for TCM, Ayurveda is not only a collection of herbal therapies, but a regimen covering the general mode of life: social conduct, behaviour and diet. In particular, the rejuvenation therapy prescribed by *Rasayana* should begin in adult life or midlife because it could not be effective when started to late, thus pointing out the preventive rather than curative efficacy of this approach [134]. Interestingly, some *Rasayana* preparations directly refer to dementia and it is remarkable that an ancient healing system has elaborated a theory and a therapy for age-related illnesses, considered as inexplicable phenomenon and attributed to spiritual sphere by contemporary ethnomedical systems. Possibly, the most important plant species in Ayurvedic life style, with reference to neuroprotection, is *Curcuma longa* L. 

### 4.1. *Curcuma longa* L.

**Figure 7:**
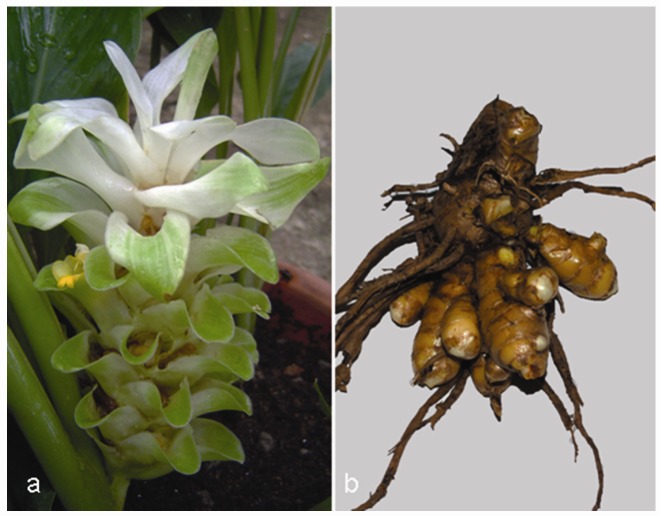
*Curcuma longa* L.: aerial parts (a) and rhizome (b).

**Figure 8:**
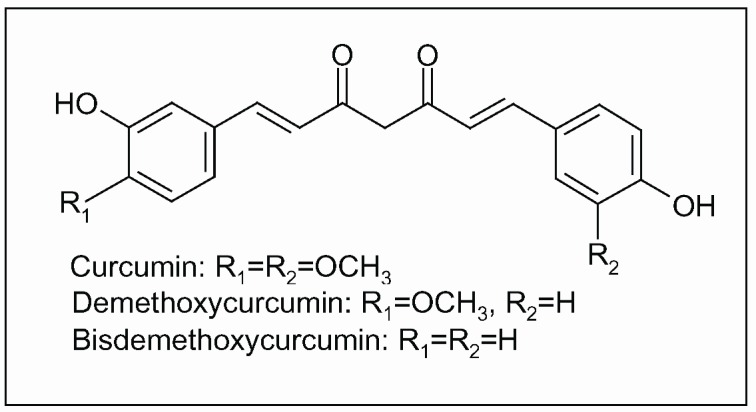
Curcuminoids, polyphenolic constituents of turmeric, the dried ground rhizome of *Curcuma longa* L.

The *Curcuma* genus belongs to the Zingiberaceae, the same family of *Zingiber *(ginger). *Curcuma longa* is a short-stemmed (~ 100 cm in height), perennial plant (Figure 7a) naturally growing throughout the Indian subcontinent and in tropical Asia, particularly in Southeast Asia. Turmeric, the dried ground rhizome of *C. longa* (Figure 7b), is a spice contained in curry, widely used for its flavouring in many food preparations and recipes. The bright yellow colour of turmeric is due to curcumin (Figure 8), the main bioactive constituent and the colouring ingredient present in the powdered rhizome. Many synonyms are known for turmeric: *haldi* in Hindi, *chiang huang* in Chinese, *ukon* in Japanese, *haridra* or *gaur*i in Sanskrit, *kurkum* in Arabic [135,136,137]. 

In addition to its applications as spice, dye, food additive and preservative in traditional Indian cookery, turmeric has been used for thousands of years in Ayurvedic and Chinese medicine. In the 12th-13th century, it was introduced, together with other spices, to Western countries by Arab traders and Marco Polo, after they visited India. Currently, turmeric continues to be widely used as an alternative medicinal agent in many parts of southern and eastern Asia, where it is considered as a ‘blood purifier’, for the treatment of common ailments such as dyspepsia, flatulence, liver disorders, arthritis, urinary tract diseases, wounds, jaundice, eye infections and skin diseases such as acnes and pemphigus [135,136,137].

The most bioactive chemical constituents of turmeric are curcuminoids, a group of polyphenols including mainly curcumin, demethoxycurcumin and bisdemethoxycurcumin (Figure 8). Components of turmeric are currently undergoing scientific evaluation for numerous potential benefits due to their anti-inflammatory, antiproliferative, pro-apoptotic, antioxidant, antiviral and antidiabetic activity. Numerous molecular targets of curcumin have been identified over the years, including cyclooxygenase (COX)-2 and lipoxygenase (LOX) [135,136,137].

Epidemiological studies have raised the hypothesis that the wide use of *Curcuma* among Indians may explain the significantly lower prevalence of AD in India compared to US [138]. In a transgenic animal model of AD, supplementation with a low dose of curcumin (160 ppm), for six months, reduced indices of both inflammation and oxidative stress. In particular, the levels of proinflammatory cytokine IL-1β, of oxidised proteins and of Aβ peptide decreased significantly [139]. Anti-amyloidogenic activity of curcumin was extensively reported in *in vitro* and animal models [140,141,142,143,144]. The binding to the redox-active metals iron and copper suggest another neuroprotective mechanism induced by curcumin, at least in animals [145]. The combination of nonsteroidal anti-inflammatory drugs (NSADs) and curcumin attenuated oxidative damage, cognitive deterioration and Aβ peptide deposition in both cell culture and animal model,. In the same study, anti-inflammatory activity of curcumin was observed, due to the inhibition of cytokine production and microglia activation and to the increase of phagocytosis index [146]. Because the process of inflammation plays a major (detrimental) role in the pathogenesis of the most chronic illnesses, including neurodegenerative diseases, the therapeutic potential of curcumin as anti-inflammatory agent in the prevention and treatment of chronic disorders has been recently highlighted [147]. In fact, as previously introduced, activation of microglial cells in CNS results in the production of pro-inflammatory mediators that propagate neuronal injury exacerbating neurodegenerative diseases. In rat, curcuminoid pigments suppressed NO production by LPS-activated microglia [148]. In a model of global cerebral ischemia, induced in Mongolian gerbils by transient occlusion of common carotid arteries, administration of curcumin by intraperitoneal injections (30 mg/kg body weight) for two months attenuated ischemia-induced neuronal death and glial activation. The decrease of lipid peroxidation, mitochondrial dysfunction and apoptotic indices were other biochemical responses mediated by curcumin. Cerebral I/R injury was also ameliorated, as shown by the locomotor activity of treated animals after I/R. Finally, bioavailability experiments reported a rapid increase of curcumin in plasma and brain tissues within 1 hour after administration [149].

## 5. Mediterranean Traditional Diets

**Figure 9:**
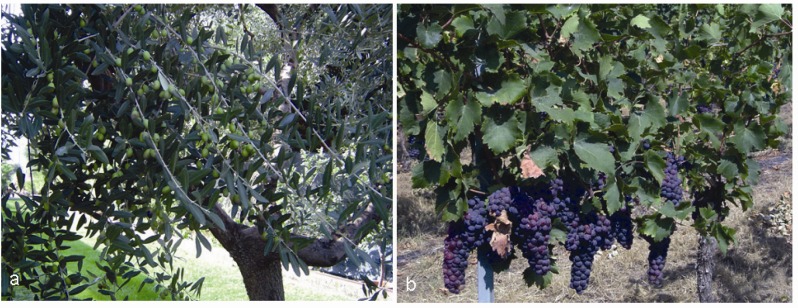
Traditional Mediterranean cultivations: *Olea europea* L. (a) and *Vitis vinifera* L. (b).

In the Mediterranean basin, different traditional dietary patterns have developed in each geographical subarea (southern Italy and France, Spain, Greece, Turkey, Northern Africa and Middle East), and, hence, a unique Mediterranean diet does not really exist. Traditionally, these dietary habits originated in areas where olive (*Olea europaea* L.) and grapevine (*Vitis vinifera* L.) (Figure 9a,b) were cultivated, olive oil and wine produced and regularly consumed [150]. Besides these foodstuffs, other main components of Mediterranean diets include whole grains, fruits, vegetables, legumes, nuts, yogurt and ricotta as dairy products, fish and white meat as protein sources [151]. Nowadays, Mediterranean diets, rich in fruits and vegetables, are correlated to a low incidence of the chronic-degenerative disorders mostly spread in western populations, and compelling evidences point out the reduced risk of cancer, cardiovascular and neurodegenerative diseases in Mediterranean populations, compared with other industrialized countries [152,153,154].

### 5.1. Grape (Vitis vinifera L.) and red wine

As attested by Theophrastus and Hesiod, viticulture and winemaking were extensively practiced in ancient Greece, though it is widely believed that both these practices began earlier, in the Neolithic Period (8,500-4,000 BC). Besides religious, social and academic (at the symposium) contexts wherein wine was introduced, its medical uses were studied by Greek physicians, as reported by Hippocrates’ studies (460-370 BC). He recommended wine to cure fever and as analgesic, antiseptic, diuretic, tonic and digestive [155]. The Romans also attributed therapeutic properties to wine, and Galen (129-200 AD), in particular, provided a detailed description on the medical uses of wine in his practice. In the care of gladiators, he used wine as antiseptic for wound healing and as analgesic for surgery. Other illnesses that Roman physicians treated by wine included depression, memory loss, constipation, diarrhoea, gout, halitosis, snakebites, tapeworms, urinary tract ailments and vertigo [156].

**Figure 10:**
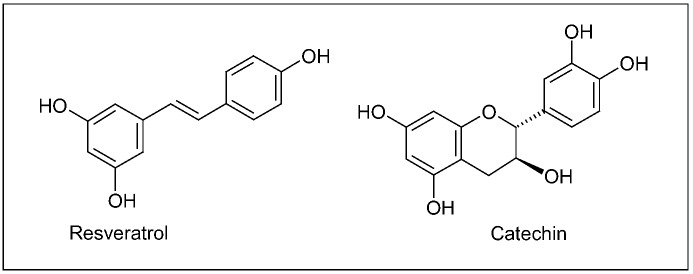
A stilbene (resveratrol) and a flavonoid (catechin) typically present in grape products.

Grape/wine chemistry is quite complex, and, in berry, bioactive secondary metabolites are mainly distributed in epidermal (skin) tissues and seeds. Phytochemicals include plenylpropanoids, isoprenoids (responsible for the wine flavouring) and alkaloids (such as indolic compounds), whose amount and variety *in planta* strictly depend on genetic, environmental and agricultural factors [157]. Though the great diversity of molecules present in grape, polyphenols represent the archetype of the health-promoting effects arising from a regular, moderate wine consumption, as attested, in the last decades, by the great amount of studies focusing on the biological activity of particular grape polyphenols, such as the stilbene resveratrol and flavonoids (Figure 10) [158,159].

As recently reviewed, many prospective, population-based cohort and case control studies have provided the substantial evidence that a regular (daily or possibly 3-4 times weekly) intake of moderate amounts (2 glasses/days) of red wine, at meals and in the context of a Mediterranean dietary style, is associated with a lower risk of developing dementia and AD [160]. In particular, the largest meta-analysis on alcohol consumption and risk of dementia suggested that low to moderate wine drinking significantly reduced by 38 and 32% the risk of dementia and AD, respectively [161]. However, it has been suggested the hypothesis that the drinker and not the drink may produce benefits, because of the healthier life-style habits of wine drinkers in relation to their beer-, spirit-drinking and abstention counterparts. Wine drinkers are more often women, college graduates, non-smokers who perform a moderate physical activity [162,163]. In any case, immoderate or excessive consumption of wine and/or other alcoholic beverages is associated with an increased risk of dementia, possibly due to the neurotoxicity of ethanol or resulting from the concomitant malnutrition/nutrition deficiency [164]. In the Copenhagen City Hearth Study, wine intake on a monthly, weekly or daily basis was associated with a lower risk of stroke, compared with no wine intake. No association between beer or spirit consumption and risk of stroke was reported, thus suggesting that some wine components, in addition to ethanol, may be responsible for the beneficial effect of wine drinking on the risk of stroke [165]. The Framingham Study evaluated the association between the type of alcoholic beverage and incidence of ischemic stroke, showing a protective effect of wine consumption among subject aged 60–69 years [166]. Results from a case-control study on young women were consistent with the above reported data and, in general, beer and spirit drinking failed to exert neuroprotective effects against ischemic stroke [167]. From the above reported and other studies, the classical J-shaped relationship between alcohol consumption and all-cause mortality could also be extend to the relationship between wine intake and risk of neurodegenerative diseases, besides cardiovascular disorders and cancer [168,169]. In J-shaped curve, regular and moderate wine drinking, at the nadir of J, is beneficial, whereas abstinence and heavy intake, on the short and long arm, respectively, are both detrimental, though to a different extent [160].

Although association does not imply causality, mechanisms (putatively) involved in wine-induced neuroprotection have been deeply investigated. In general, protective effects of grape polyphenols against neurodegenerative diseases can be ascribed to their anti-amyloidogenic, antioxidant and anti-inflammatory activity [170,171,172,173,174]. The daily administration of resveratrol (50 or 100 mg/kg) for 1 or 2 weeks to adult male mice significantly prevented the nigrostriatal dopaminergic neuron depletion, after the acute treatment with the neurotoxin MPTP injected itraperitoneally [175]. In different cell lines stably transfected with human APP, resveratrol was shown to promote the intracellular degradation (clearance) of Aβ peptides via a mechanism that involves the proteasome, without direct inhibition of the enzymes β- and γ-secretases implicated in the Aβ protein synthesis [176]. Neuroprotective effects of three major grape polyphenolic constituents (resveratrol, quercetin and catechin, Figure 2,10) were assessed in cultured mixed (glial/neuronal) cells of rat hippocampus, as previously introduced a brain area severely affected in both AD and ischemia. Hippocampal cell treatment with polyphenols reduced the cytotoxicity induced by both the NO free radical donor sodium nitroprusside (SNP) and intracellular ROS accumulation [177]. In a mouse model of AD, the moderate consumption of Cabernet Sauvignon promoted the non-amyloidogenic processing of APP mediated by α-secretase, thereby preventing or delaying the generation of Aβ peptides [178]. More recently, a grape seed polyphenolic extract significantly prevented Aβ protein oligomerization, by inhibiting the Aβ protein aggregation into high-molecular-weight oligomeric Aβ species, both *in vitro* and in Tg2576 mice. Besides, when orally administered to these animals, the extract attenuated the cognitive deterioration typical of AD [179]. 

Other mechanisms by which polyphenols retard the ageing process and delay the onset of ageing-related diseases resemble those induced by caloric restriction (CR), suggesting that these compounds and CR share quite similar molecular pathways [180,181]. A moderate reduction in calorie intake of 20–40% significantly extends lifespan in a wide spectrum of organisms, ranging from bacteria to primates, a process mediated by a family of nicotinamide adenine dinucleotide (NAD+)-dependent deacetylases, the sirtuins (from silent information regulator, SIR, proteins) [182,183,184]. In mammalians, sirtuins represent novel therapeutic targets to treat age-associated and neurodegenerative diseases, being implicated in a variety of cellular functions, ranging from gene silencing, over the control of cell cycle and apoptosis, to mitochondrial function and energy metabolism [185,186,187]. Additionally, these proteins play a role in protecting neurons against damage and in prolonging their survival in AD [188,189]. Therefore, sirtuin activating compounds (STACs) represent a promising class of therapeutics, including resveratrol, quercetin and other polyphenols able to activate SIRT1. The latter deacetylates both histones and non-histone substrates, such as the tumor suppressor protein p53 and the transcription factor NF-kB, thus regulating some pathophysiological processes affected during ageing and modified by CR [190,191]. 

### 5.2. Salvia officinalis L.

**Figure 11:**
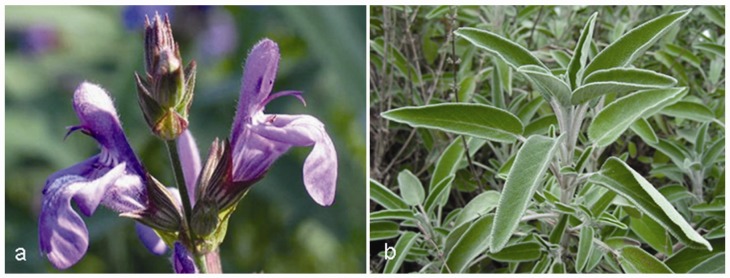
*Salvia officinalis* L.: flowers (a) and leaves (b).

*Salvia* is an important genus in the Lamiaceae family consisting of around 900 species, and some species have been cultivated worldwide for medical and culinary uses (Figure 11a,b). *Salvia* derives from Latin *salvus*, meaning ‘healthy’, suggestive of the miraculous therapeutic properties attributed to this plant by traditional medicine, and the textual sources concerning the cultivation and the use of *Salvia* in the Mediterranean area date back to the ancient Egyptian, Greek and Roman civilizations. Though it is not known which species was used, sage was listed in the Ebers Papyrus (1,500 BC) as a remedy for itching. Followers of Hippocrates praised its styptic and strengthening qualities as well as its beneficial effects on menstruation. Under the name of *Salvia*, the plant was described in the works of the Romans Plinius, Dioscorides and Galen. They recommended it for warming and contractions, for coughs, hoarseness, for labour pains and ulcers [192]. At the end of 17th century, the physician and botanist Tabernaemontanus recommended many sage preparations from different geographical origins for the treatment of a ‘weak brain’ and to strengthen memory [2].

**Figure 12:**
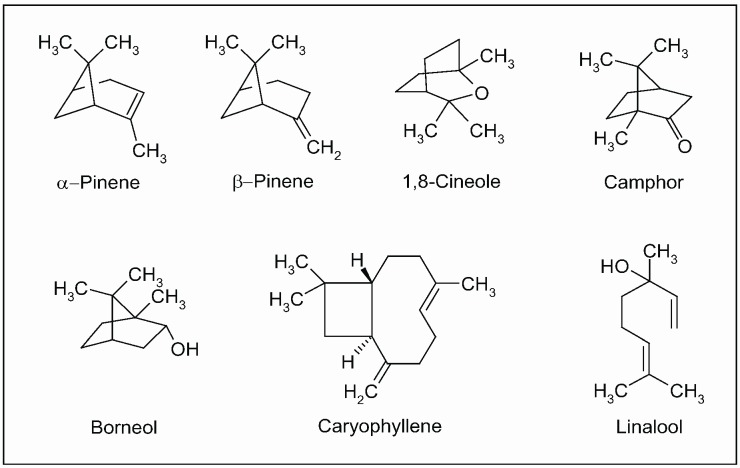
Main isoprenoids (mono- and sesquiterpenes) present in essential oil extracted from aerial parts of *Salvia officinalis* L.

Currently, the health benefits of the Mediterranean diet have also been attributed to the regular consumption of traditional spices, considered as significant sources of bioactive phytochemicals [193,194,195,196,197]. In particular, many pharmacological studies on sage has been performed on volatile constituents (essential oils). The *in vitro* anticholinesterase activity of *S. officinalis* and *S. lavandulaefolia* (Spanish sage) was reported in both human erythrocytes and brain tissues (post-mortem) [198,199]. This activity was attributed to the synergy among monoterpenes present in essential oil, mainly α- and β-pinene, 1,8-cineole, camphor, borneol, caryophyllene and linalool (Figure 12). Additionally, pure compounds from the extract were tested, none of which fully accounted for the activity of the essential oil [198,200,201].

It is noteworthy that the constituents of an essential oil vary within the genotype, geographic distribution and environmental factor. In a clinical trial, a standardized essential oil extract of *S. lavandulaefolia* enhanced memory in young adult volunteers by improving the immediate word recall [199]. The same authors reported that an acute dose of *S. lavandulaefolia* essential oil positively modulated mood and cognitive performance in healthy young adults [202]. Besides inhibition of cholinesterase, other properties of sage essential oil, mainly antioxidant and anti-inflammatory activity, in part due to polyphenols and other phenylpropanoids, are currently considered relevant to the symptomatic treatment of AD [203,204,205]. In a double blind, randomized, placebo-controlled trial, the efficacy of *S. officinalis* extract was evaluated in patients with mild to moderate AD. After 16 weeks of administration, the treatment group experienced a significantly better outcome on cognitive function than placebo and no difference between the two groups were observed in terms of safety and side-effects. Though the small number of participants and the relatively short follow up, this clinical investigation suggests that sage may have potential for treatment of AD and related disorders [206].

## 7. Two international beverages: coffee and tea

### 7.1. Coffee (Coffea spp.)

**Figure 13:**
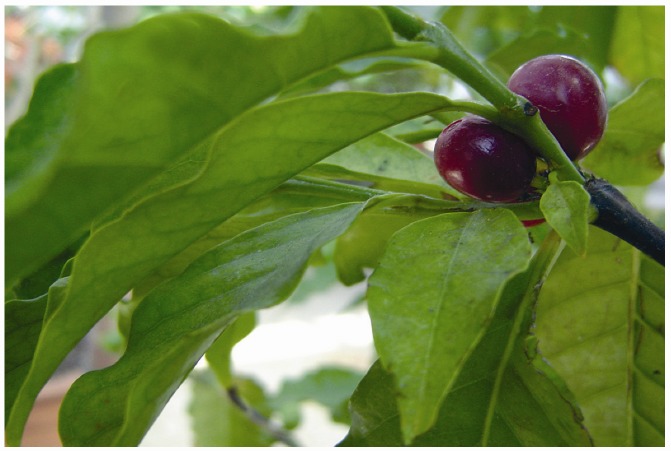
*Coffea arabica* L.: leaves and fruits (coffee cherries).

Native to Yemen and Ethiopia, the genus *Coffea* (Rubiaceae family) includes two main species: *C. arabica *L. (Figure 13), which accounts for most of the world coffee production, and *C. canephora* L. (syn. *C. robusta* L.). Coffee is the brewed beverages prepared from roasted seeds, also called coffee beans, the most frequently consumed stimulant drink all over the world. According to 1998 data, the highest annual coffee consumption per capita was observed in Scandinavian countries (greater than 10 kg of coffee beans), whereas, in US, consumption was about 4.7 kg/person/year, that is approximately 200 mg of caffeine or two cups of coffee per person per year [207].

**Figure 14:**
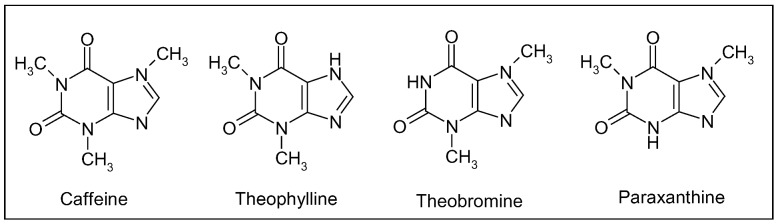
Caffeine and structurally related methylxanthines found primarily in tea (theophylline) and chocolate (theobromine); paraxanthine is the main hepatic metabolite of caffeine after coffee ingestion.

Caffeine, a methylxanthine (Figure 14), is the most important bioactive constituent of this beverage providing neuroprotection. Other structurally similar xanthine alkaloids are theophylline and theobromine (Figure 14), found primarily in tea and chocolate, respectively. Tea is prepared from the leaves of *Camellia sinensis* Kuntze (Theaceae family), also native to the Old World, whereas cacao derives from the seeds of the New World species *Theobroma cacao* L. (Sterculiaceae), used by the Aztecs as medicine. These three compounds arise from purine and differ structurally in the number and position of methyl groups: caffeine possesses three groups at positions 1, 3 and 7, whereas theophylline and theobromine are dimethyl xanthine isomers with methyl groups at position 1,3 and 3,7, respectively (Figure 14). Other important sources of methylxanthine alkaloids are kola nuts (genus *Cola*, Sterculiaceae family) native to Tropical Africa and used to flavour carbonated beverages, guaraná (genus *Paullinia*, Sapindaceae) from South America and a popular beverages in Brazil, and Mate (genus *Ilex*, Aquifoliaceae) native to southern South America and the national drink of Argentina [3]. 

As regards pharmacological activity, methylxanthines act as adenosine-receptor antagonists. In particular, caffeine is a nonspecific, competitive blocker of adenosine A1 and A2A receptors, distributed throughout the central nervous system [208]. Binding of adenosine to target receptors has general depressant effects, slowing hearth rate and lowering blood pressure. The half-life of caffeine is 3–7 h in adults and, after ingestion, it is metabolized by the hepatic cytochrome p450 1A2 enzyme into three primary metabolites: paraxanthine (1,7-dimethylxanthine, *ca.* 80%), theobromine (*ca.* 10%) and theophylline (*ca.* 5%) (Figure 14) [209].

According to human epidemiological studies, caffeine, as well as other adenosine A2A receptor antagonist, may play a role in preventing or delaying the onset of AD. A case control study involving subjects aged 50 years with probable diagnosis of AD and sex-matched controls found that individuals consuming 2 cups of coffee (approximately 200 mg of caffeine) per day for the previous 20 years were at a significantly lower risk of developing the disease than those that consumed less caffeine [210]. A meta analysis of two case control and two prospective studies examining the effects of coffee on AD also reported that this beverage was protective [211]. In Cancer Prevention Study II Cohort, a prospective study involving more than one million of Americans, coffee intake was reported to be inversely associated with PD mortality in men and in women who did not use postmenopausal estrogen therapy [212]. These results were in accordance with previous studies in which coffee consumption was consistently protective against PD for men and women in the absence of estrogen therapy [213,214].

It is noteworthy that acute intake of high doses of coffee (5 cups of coffee, approximately 500 mg of caffeine, at one sitting) results in activation of stress responses, as demonstrated by increased plasma levels of cortisol, β-endorphin and epinephrine, in turn raising heart rate, blood pressure and releasing free fatty acids from storage [215].

### 7.2. Tea (Camellia sinensis Kuntze)

**Figure 15:**
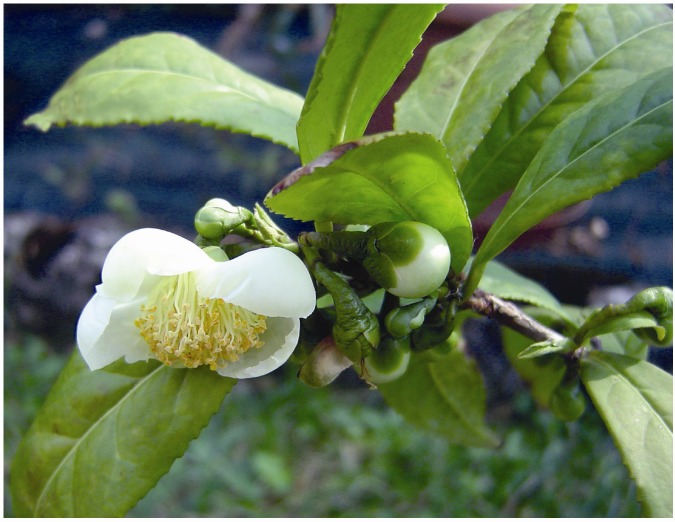
*Camellia sinensis* Kuntze: leaves and flowers.

**Figure 16:**
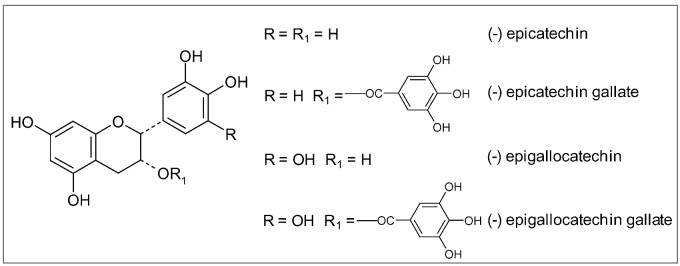
Chemical structures of major catechins.

All tea plants cultivated in different regions of the world belong to the same species, *Camellia sinensis* Kuntze, though the local growing conditions, (altitude, climate, soils, *etc*.) may produce numerous distinctive leaves (Figure 15). Nevertheless, the way the leaves are processed is even more important in determining the characteristics of the three predominant types of tea: green, black and oolong. Green tea is the least processed and thus provides the most antioxidant polyphenols, particularly catechins (epigallocatechin, epigallocatechin-3-gallate), flavonols (myricetin, quercetin, kaempherol) and proanthocyanidins [216]. Being catechins (Figure 16), particularly epigallocatechin-3-gallate (EGCG), 10–20 times more concentrated than flavonols in normally brewed tea [217], they seem to be responsible for most of the health benefits linked to this beverage. Green tea is made by briefly steaming the freshly harvested leaves, in order to softening them and preventing their fermentation and color changing. After steaming, the leaves are rolled, then spread out and dried with hot air until they are crisp. The resulting greenish-yellow tea has a green, slightly astringent flavor close to the taste of the fresh leaf. 

Most of the research showing the health benefits of green tea is based on the amount of green tea typically consumed in Asian countries, that is to say about three cups per day, which would provide 240–320 mg of polyphenols [218,219,220]. Just one cup of green tea supplies 20–35 mg of EGCG, which has the highest antioxidant activity of all the green tea catechins [216]. Green tea drinkers appear to have lower risk for a wide range of diseases, from simple bacterial or viral infections to chronic degenerative conditions including cardiovascular disease, cancer, stroke, periodontal disease, and osteoporosis [221,222,223,224,225,226,227,228,229,230]. 

As regards protection against AD and PD, green tea catechins, until recently thought to work simply as antioxidants, are now known to invoke a wide spectrum of neuroprotective cellular mechanisms. These include iron chelation, scavenging of free radicals, activation of signaling pathways, and regulation of mitochondrial function to avoid excessive production of free radicals [231,232,233]. As reported above, iron accumulation in specific brain areas and free radical damage to brain cells are considered the major damaging factors responsible for a wide range of neurodegenerative disorders including AD and PD. In the brain, epigallocatechin-3-gallate (EGCG) has been shown to act as an iron chelator, binding to and removing iron, thus preventing it from contributing to the production of free radicals. In addition to removing iron, EGCG also increases the activity of two major antioxidant enzymes, superoxide dismutase (SOD) and catalase, further helping to decrease free radical damage [233]. Another active compound in green tea, epicatechin (EC) (Figure 16), reduces the formation of β-amyloid protein and of the consequent plaque-like deposits in the brain characteristic of AD [77]. The protective effects of black and green tea extracts and their main constituents, epigallocatechin gallate and epicatechin gallate have been shown in an in vitro system of cultured neurons. In presence of these extracts neurons survived to the toxic effect of β-amyloid protein [232]. Green tea polyphenols have also demonstrated the ability to affect cell signaling pathways, in particular the MAPK pathways, which are triggered by oxidative stress. MAPK signaling pathways in brain cells are thought to play a critical role in neurodegenerative diseases [231]. 

Although no human studies on AD have yet reported benefit from tea consumption, recent population studies have shown that simply consuming two or more cups of green tea daily reduces risk of cognitive decline and PD. In a cohort study at Japan's Tohoku University, using a Mini-Mental State Examination (a well-accepted standardized test for measuring cognitive function) on 1,003 subjects over age 70, researchers showed that drinking more than two cups a day of green tea reduces chances of cognitive impairment in both men and women by 64% [234]. And at every level of cognitive impairment, from minimal to severe, those drinking the most green tea experienced significantly less mental decline than those drinking the least. In particular, compared with elderly Japanese who drank less than three cups a week, those drinking more than two cups a day had a 54% lower risk of age-related declines in memory, orientation, ability to follow commands and attention. Those drinking four to six cups of green tea a week (one cup a day) had a 38% lower risk of declines in brain function [234]. 

As reported in the previous section, tea contains also caffeine, although half that found in coffee [216]. The amount of caffeine that ends up in a cup of green tea varies according to the amount of tea used and the length of time the leaves are infused [216,217]. Most of the caffeine in green tea is extracted into the water the first time the tea is infused. There is limited research in the published literature comparing the caffeine content of green vs. black tea. A recent study measured the caffeine content in the dry matter of the tea leaves, an approach that allows for control of any confounding variables related to preparation techniques that may impact the caffeine content in the final tea product [216]. This study found that the caffeine content of one gram of black tea ranged from 22–28 milligrams while the caffeine content of one gram of green tea ranged from 11–20 milligrams, reflecting a significant difference. Interestingly, at least two beneficial components in green tea, catechins and the amino acid L-theanine, lessen the impact of its caffeine. When green tea is brewed, its caffeine combines with catechins in the water, reducing the caffeine's activity compared to coffee or cocoa [235]. In addition, L-theanine, which is only found in tea plants and some mushrooms, directly stimulates the production of alpha brain waves, calming the body while promoting a state of relaxed awareness [235] *.*

## 7. Conclusions

Plants, in the form of herbs, spices and foods, constitute an unlimited source of molecules available for improving human health. Nonetheless, a single plant contains hundreds or thousands of secondary, bioactive metabolites, a chemical diversity that determined the evolutionary success of plants, favouring their adaptation to a changing environment [216]. In this view, to ascribe the health-promoting effects of a medicinal herb or a plant food only to a molecule, or a single class of compounds, represents an inappropriate and inopportune task. It is likely that different phytochemicals produce *in vivo* additive and/or synergistic effects, thus amplifying (or reducing/inhibiting) their activities.

As implicitly outlined in this survey, most of our current knowledge about CNS-active plants of cultural and traditional importance arose from ethnobotanical and ethnopharmaceutical (including historical) studies, as for other natural active ingredients. Some famous examples include *Atropa belladonna* L. and *Hyoscyamus niger* L. from Eurasia for atropine and hyoscyamine respectively, *Erythroxylum coca* Lam. from Central America for cocaine, *Papaver somniferum* L. for codeine and morphine and *Ephedra sinica* Stapf. for ephedrine, both from Asia. Therefore, the ethnobiological approach represents a powerful tool for the discovery of new neuroactive natural products from plants used as medicinal herbs, spices or food in different cultural (ethnic) groups. 

A better comprehension of the bioavailability of dietary phytochemicals is critical in order to correctly evaluate their bioactivity, to interpret the experimental results and to design new approaches, particularly in CNS. However, biokinetic data supporting their absorption, distribution, metabolism and excretion in human body are still fragmentary, despite the enormous amount of indications on their bioactivities. Dietary phytochemicals have to be absorbed to exert their health benefits, and human studies indeed reported the direct evidence of the absorption and urinary excretion of these compounds after the intake. Moreover, for a suitable neuroprotective agent, a very important property regards its ability to cross the blood-brain barrier (BBB), in order to reach the target sites of the CNS. A limited number of studies, both *in vitro* and on animal models, focused on the ability of flavonoids to cross the endothelial cell layer of BBB, depending on the compound lipophilicity and on the activity of specific transporters [217,218,219,220,221,222]. It has been suggested that polyphenols may interact with plasma membrane transporters or receptors, triggering downstream intracellular signaling pathways. At present, only a receptor for EGCG has been identified on vascular cells [223]. Nevertheless, the high variability of the data concerning the bioavailability, at least for polyphenols, depends on the complexity of food matrixes (fruits and vegetables) and on the chemical structure of the specific compounds [224,225,226].

Finally, though the presence of receptors or transporters for polyphenols or other phytochemicals in brain tissues remains to be ascertained, compounds with multiple targets appear as a potential and promising class of therapeutics for the treatment of diseases with a multifactorial etiology [227,228,229].
